# Fresh Azolla, *Azolla pinnata* as a Complementary Feed for *Oreochromis niloticus*: Growth, Digestive Enzymes, Intestinal Morphology, Physiological Responses, and Flesh Quality

**DOI:** 10.1155/2023/1403704

**Published:** 2023-01-30

**Authors:** Mohamed M. Refaey, Ahmed I. Mehrim, Osama A. Zenhom, Hamada A. Areda, Janice A. Ragaza, Mohamed S. Hassaan

**Affiliations:** ^1^Animal Production Department, Faculty of Agriculture, Mansoura University, PO 35516 Al Mansoura, Egypt; ^2^Central Laboratory for Aquaculture Research, Agriculture Research Center, Abbasa, Abou Hammad, Egypt; ^3^Animal Production Department, Faculty of Agriculture, Damietta University, Egypt; ^4^Ateneo Aquatic and Fisheries Resources Laboratory, Department of Biology, School of Science and Engineering, Ateneo de Manila University, Katipunan Ave., Loyola Hts., Quezon City, NCR, Philippines 1108; ^5^Animal Production Department, Faculty of Agriculture, Benha University, Benha, Egypt

## Abstract

Azolla is a potential fish feed ingredient due to its high nutritional value, abundant production, and low price. This study is aimed at evaluating the use of fresh green azolla (FGA) as a replacement ratio of the daily feed intake on the growth, digestive enzymes, hematobiochemical indices, antioxidant response, intestinal histology, body composition, and flesh quality of monosex Nile tilapia, *Oreochromis niloticus* (with an average initial weight of 108.0 ± 5.0 g). Five experimental groups were used and differed in commercial feed replacement rates of 0% (*T*_0_), 10% (*T*_1_), 20% (*T*_2_), 30% (*T*_3_), and 40% (*T*_4_) with FGA for 70 days. Results showed that 20% replacement with azolla gave the highest values of growth performance and hematological parameters and the best feed conversion ratio, protein efficiency ratio, and fish whole body protein content. The highest levels of intestinal chymotrypsin, trypsin, lipase, and amylase were noted in 20% replacement with azolla. Fish fed diets with FGA levels of 10% and 40% showed the highest values for the thickness of the mucosa and submucosa layers among all treatments, respectively, while the length and width of the villi decreased significantly. No significant (*P* > 0.05) differences in the activities of serum alanine transaminase, aspartate transaminase, and creatinine were detected among treatments. The hepatic total antioxidant capacity and the activities of catalase and superoxide dismutase significantly (*P* < 0.05) increased while the activity of malonaldehyde decreased with increasing the replacement levels of FGA up to 20%. With increasing levels of dietary replacement with FGA, muscular pH, stored loss (%), and frozen leakage rate (%) were significantly decreased. Finally, it was concluded that the dietary replacement of 20% FGA or less may be considered a promising feeding protocol for monosex Nile tilapia, which may lead to high fish growth, quality, profitability, and sustainability for the tilapia production sector.

## 1. Introduction

Fish feed cost accounts for up to 60% of total fish production [1, 2]. Fishmeal, soybean, and corn are among the feed ingredients with supply and high pricing difficulties, which have led to an increase in the price of fish feed [3]. Dietary protein quality and quantity have a significant impact on the health and growth performance of fish [4]. Researchers have employed a variety of strategies to reduce the cost of feed during the past few decades, mainly by manipulating the type of protein, which is thought to be the most expensive component. These strategies included using less expensive plant protein sources like cottonseed meal and rapeseed meal [5, 6], as well as less conventional protein sources such as poultry by-product meals [7], blended alternative proteins [8], black soldier fly larvae meal [9], and methanotroph bacteria meal [10]. In the same perspective, several supplements such as organic acids [11], taurine [12], and a combination of taurine and methionine have been used to act as growth stimulants that boost the value of dietary protein [13].

In the same light, fish like tilapia have also been fed a variety of aquatic weeds, such as *Salvinia molesta*, *Lemna minor*, and azolla *Azolla pinnata* [14]. Aquatic azolla plants are cultured in tropical and subtropical habitats [15]. According to research, azolla is regarded as a crucial feed ingredient or feed supplement for a variety of farm animals including fish due to its high nutritional value and protein content [16]. In addition, Azolla contains nearly all the necessary amino acids and has a crude protein content of 19–30% on a dry-weight basis [17]. Roy et al. [18] found Azolla to contain up to 18 amino acids including glutamic acid (12.6% protein), aspartic acid (9.3%), leucine (8.4%), alanine (6.4%), arginine (5.9%), glycine (5.6%), and valine (5.5%). Additionally, it is high in minerals such as iron, calcium, magnesium, potassium, phosphorus, and manganese, as well as vitamins like vitamin A, vitamin B12, and beta-carotene and some probiotics and biopolymers [19, 20].

Azolla can be used as a potential diet for fish as it is easy to grow, has a high yield, and is inexpensive to produce, which is reflected in its lower price compared to the price of a kilogram of fish feed. According to Santiago et al. [21], Nile tilapia fry fed rations containing up to 42% of azolla outperformed fish on a fish meal-based control diet, demonstrating the beneficial effects of feeding azolla. Young Nile tilapia use azolla meal more effectively than adults [22]. Moreover, Magouz et al. [23] reported that the ideal inclusion levels of azolla meal in the diet of Nile tilapia ranged from 10 to 20%. However, another study [24] found that as the level of azolla inclusion in the experimental meals went from 0 to 50%, the final mean weight of Nile tilapia declined. Azolla (15%) and *Arthrospira platensis* (3%) meals were recommended [25] as a partial replacement for fishmeal in semi-intensive feeding. Another study [26] recently concluded that supplementing *Spirulina platensis* (1%) and *Azolla nilotica* (5%) is advantageous for enhancing Nile tilapia growth performance. The same trend was observed in different fish species such as common carp (*Cyprinus carpio*) fed azolla that substituted 15% of soybean meal [27], fringed-lipped peninsula carp *Labeo fimbriatus* fed azolla up to 40% [28], and Thai silver barb (*Barbonymus gonionotus*) fed 25% azolla in the diets [29] without reducing flesh quality and feed cost savings.

Nile tilapia is the most widely cultured fish with an annual growth rate of 8% [3]. In Egypt, Nile tilapia is also ranked first in the total fish production among other fish species [30]. This may be due to their ability to feed on a wide variety of feeds, fast growth rates, high feed conversion ratios, and capacity to reproduce in captivity [31]. The most recent studies used azolla in dried form for feeding fish. It is rarely used in fresh form or for feeding adult fish. Therefore, the aim of the current study is to evaluate the effects of the quantity replacement of fresh green azolla (FGA) as a daily feed on growth performance, nutritional efficiency, digestive enzymes, intestinal morphology, hematobiochemical indices, antioxidant responses, and flesh quality of adult Nile tilapia males after 70 days of feeding.

## 2. Materials and Methods

### 2.1. Azolla Cultivation

Azolla was obtained from a private farm in Mansoura City, Dakahlia Government, Egypt. Two ponds (length × width × height: 6 m × 2 m × 0.5 m; total volume of 6 m^3^) were prepared for azolla cultivation. The pond floor was covered with a thin layer of clay that had been mixed with compost to a depth of 10 cm. The ponds were then submerged to a depth of 40 cm of water. The green azolla begins to sprout in the ponds after 15 days of culture. To lower its moisture content, FGA was harvested daily 24 hours before feeding the fish in the amount needed to feed Nile tilapia. Chemical fertilizer such as calcium phosphate (12.5% P) was supplied to the pond at a rate of 20 g every 15 days while the crop was being grown. Using the methods of AOAC [32], the proximate chemical composition of FGA was estimated and is displayed in Table 1.

### 2.2. Feeding Procedure

**Figure 1:**
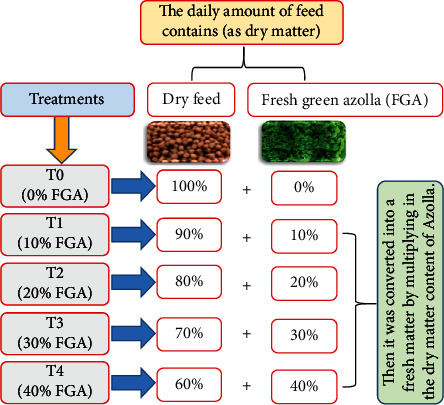
Schematic diagram of the experimental design.

The feeding trial was carried out at the Fish Research Unit of the Faculty of Agriculture, Mansoura University, Egypt. Monosex all-male Nile tilapia with an average initial body weight of 108 ± 1.45 g were acclimated to the lab conditions for 15 days before fed the commercial basal diet (BD) twice daily at 9:00 a.m. and 2:00 p.m. Fish were stocked at a density of 15 fish tank^−1^ (1 m^3^ tank volume) after the acclimation period and randomly assigned to five experimental treatments, each with three replicates. An air stone connected to an air pump was placed inside each tank (Super Pump, SP-780, 5 W, 3.5 L min^–1^). The five experimental treatments (*T*_0_ to *T*_4_) were designed as different quantity replacement rates 0% (*T*_0_; control), 10% (*T*_1_), 20% (*T*_2_), 30% (*T*_3_), and 40% (*T*_4_) of FGA (Figure 1). The replacement levels were based on the protein %, which is the amount of the actual commercial feed that needed to be replaced with FGA on a dry basis. For FGA protein to reach the protein level of the commercial feed, the feed percentages of the treatments were created. For the first six weeks, fish were fed at a rate of 3% of their live body weight and subsequently at 2% until to the end of experiment. FGA was supplied once daily at 12 a.m. while experimental diets were handed out twice daily at 9.00 a.m. and 3.00 p.m. Biweekly adjustments were made to the feed amount based on the variations in the actual fish body weight.

Water temperature, dissolved oxygen (DO), pH, and total ammonia nitrogen (TAN) were tested biweekly as indicators of water quality. The temperature was set at 25–26°C. DO averaged 5.65 ± 0.5 mg L^−1^ (Jenway Ltd. Model 970-DO meter, Staffordshire, ST15 0SA, UK). The pH value was 7.77 (Jenway Ltd., Model 350-pH-meter, Staffordshire ST15 0SA, UK). Using the colored approach resulting from direct Nesslerization methods and Chemists® test kits (CHEMETRICS, INC., USA) [33], TAN ranged from 0.09 to 0.02 mg L^−1^. For ten weeks, fresh groundwater was added to each tank thrice a week and replaced around 20% of the water volume. According to Bhatnagar and Devi [34], these water quality parameters are within the acceptable levels for Nile tilapia rearing.

### 2.3. Measurements

#### 2.3.1. Growth Performance, Feed Efficiency, and Body Indices

The fish were weighed at the end of the study to determine the final fish weight (g) and other growth performance metrics using the following equations:

(1) $$\begin{array}{c} \text{Total weight gain}\left(\text{TWG},\text{g}\right)=\text{FW}-\text{IW} \end{array}$$

- Average daily gain (*ADG*, *g* *fish*–1 *day*–1) = *TWG*/*T*
- Specific growth rate (*SGR*, %*day*–1) = [(ln*FW*–ln*IW*)/*T*] × 100
- Feed conversion ratio (FCR) = FI/TWG
- Protein efficiency ratio (PER) = TWG/PI

where FW is the final weight (g), IW is the initial weight (g), T is the experimental period (day), FI is the feed intake (g), and PI is the protein intake (g).

Fish (*n* = 9 per treatment) were individually weighed and had their total body length (TBL) measured, after which the liver and stomach were removed and the hepatosomatic index (HSI) and stomach somatic index (SSI) were calculated using the following equations:

(2) $$\begin{array}{c} \text{HSI }\left(\%\right)=\frac{\left[\left(\text{Liver weight}\left(\text{g}\right)\times 100\right)\right]}{\text{Fish weight }\left(\text{g}\right)},\\ \text{SSI}\left(\%\right)=\frac{\left[\left(\text{Stomach weight }\left(\text{g}\right)\times 100\right)\right]}{\text{Fish weight }\left(\text{g}\right)}. \end{array}$$

Additionally, the length of the intestine was individually measured to find the relationship between intestine length (IL, cm) and TBL (cm) and to calculate the relative gut length (RGL) according to the following equation:

(3) $$\begin{array}{c} \text{RGL}=\frac{\text{IL}}{\text{TBL}}. \end{array}$$

#### 2.3.2. Digestive Enzymes

Fish (*n* = 5 per treatment) were slaughtered after blood was collected. The middle portion of the intestine was removed and preserved at -20°C until the digestive enzymes were identified using the techniques described by Mohammady et al. [35].

#### 2.3.3. Blood Samples

The fish were starved for 24 hours before blood samples were taken. Three drops of the commercial clove oil extract dissolved in 10 L of tap water were used to anaesthetize fish (*n* = 6 per treatment). Whole blood samples were taken from the caudal peduncle and then placed in tiny plastic vials containing heparin to determine the hematological parameters. Additional blood samples were taken to obtain the serum by centrifuging the blood without heparin at 3500 g for 20 minutes, then stored in a deep freezer (-20°C) until biochemical analysis.

*(1) Hematological Parameters*. Red blood cells (RBCs), hemoglobin (Hb), mean corpuscular volume (MCV), mean corpuscular hemoglobin concentration (MCHC), platelets (PLT), packed cell volume (PCV), and total white blood cells (WBCs) were all measured in the whole blood samples. The measurement of Hb (mg dL^−1^) was done using commercial colorimetric kits (Diamond Diagnostic, Egypt). Using the methods of Dacie and Lewis [36], PLT (>103 mm^−3^) and RBCs (>106 mm^−3^) were counted using an Ao Bright-Line hemocytometer model (Neubauer Enhanced, Precicolor HBG, Germany). MCV and MCHC (%) were computed using the prescribed methods of Beutler et al. [37] while PCV (%) was measured based on the methods of Stoskopf [38].

*(2) Serum Biochemical Traits*. Utilizing commercial kits (Diagnostic System Laboratories, Inc., USA), serum biochemical components such as alanine transaminase (ALT), aspartate transaminase (AST), uric acid (UA), creatinine, total protein (TP), albumin (ALB), and globulin (GLB) were calorimetrically evaluated. TP (g dL^−1^) and ALB (g dL^−1^) were measured according to McGowan et al. [39] whereas serum ALT (U L^−1^) and AST (U L^−1^) were assessed in accordance to the methods of Henry [40]. Serum GLB (g dL^−1^) were calculated using the variations between TP and ALB. Triiodothyronine (T3, ng dL^−1^) and thyroxine (T4, g dL^−1^) serum concentrations were also measured using the Cobas 6000 immunoassay analyzer test and commercial RIA kits (Roche, Basel, Switzerland).

#### 2.3.4. Antioxidants Determination

Fish liver samples (*n* = 5 per treatment) were removed and stored at –20°C until the antioxidant activities were determined. Antioxidant parameters were estimated according to methods of Mohammady et al. [35].

#### 2.3.5. Histological, and Histometric Examination of the Intestine

Fish (*n* = 5 per treatment) were slaughtered and the middle portion of the gut was taken out for histological and histometric examination. Before being treated on slides, which included washing, dehydrating with various strengths of alcohol, clarifying with xylene, and embedding in paraffin wax, the intestines were first rapidly fixed in a 10% neutralized formalin solution. Following the methods of Roberts [41], the wax blocks were sectioned to 5 *μ*m and stained with hematoxylin and eosin to prepare the histological slides. Using a Leica DM 500 phase-contrast microscope and an ICC50W camera, photomicrographs of the histological structure were made at magnifications of ×100 (bar = 100 m). The histometric characteristics of the intestine including muscular thickness, submucosal thickness, villi length, and villi thickness were measured following the methods of Radu-Rusu et al. [42]. The lumen was measured by taking several photomicrographs at magnifications of ×40. All histometric measurements were measured by ImageJ software (National Institutes of Health and the Laboratory for Optical and Computational Instrumentation. LOCI, University of Wisconsin, USA).

#### 2.3.6. Chemical Composition of the Fish Whole Body and Fillet

The fish whole body (*n* = 3 per treatment) and muscle tissue (*n* = 9 per treatment) were removed at the end of the experiment and stored at -20°C for proximate analysis. Moisture, crude protein, crude fat, and ash contents were all analyzed in accordance to the AOAC guidelines [32].

#### 2.3.7. Flesh Quality Measurement

Fish muscle (*n* = 6 per treatment) was taken and mashed with distilled water to obtain an extract for pH measurement (Jenway Ltd., Model 350-pH-meter, Staffordshire ST15 0SA, UK). Individual dorsal muscle fillet portions were separated, weighed individually, and placed in plastic bags. Dorsal muscle samples (*n* = 12 per treatment) were kept at 4°C and -20°C for 24 hours to calculate the stored loss (SL) and the frozen leakage rate (FLR), respectively. Following Lingqiao et al. [43], SL and FLR were calculated as the percentage of initial weight dropped. Additional samples of the dorsal muscle (*n* = 6 per treatment) were held at 4°C for 72 hours to determine the drip loss (DL), which is defined as the percentage of original weight lost [44].

### 2.4. Statistical Analysis

Statistical analysis software (SAS®) version 9.1.3 for Windows [45] was used to examine all the data in this study. Prior to analysis, percentage data was converted using arcsine. Tukey's post hoc test was used after the procedure of least squares to analyze the variations between the treatments in general linear models (GLM) [46]. At a *P* value of 0.05, the statistical differences were evaluated. Polynomial regressions were performed for each response variable using the mean. Regression analysis was performed with graphic software Sigma Plot version 8 (SPSS Inc. Chicago, IL, USA).

## 3. Results

### 3.1. Growth, Feed Efficiency, and Body Indices

**Figure 2:**
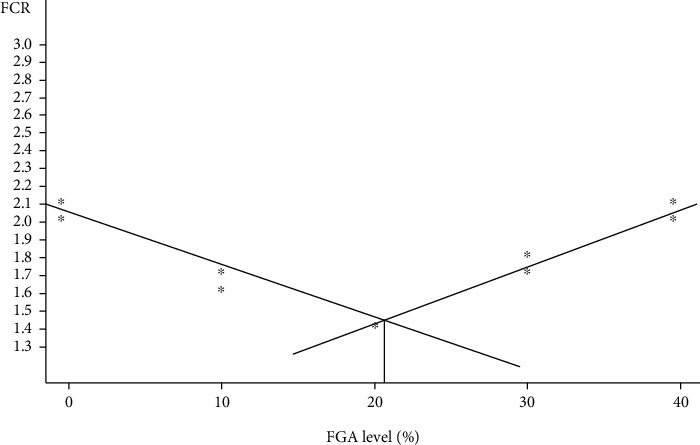
Broken-line regression analysis of FCR of Nile tilapia fed FGA with different levels. *R*^2^ = 0.931. The breakpoint is 23% g of FGA.

There was a gradual increase in FW, TWG, ADG, and SGR of monosex Nile tilapia with increasing replacement levels of FGA from the daily feed amount up to 20% (*T*_2_) (Table 2). The same parameters decreased above the 20% replacement level (*P* < 0.05). Data showed that replacing FGA at 20% of the daily feed amount gave the highest values of FW, TWG, ADG, and SGR as well as the best values of FCR and PER among the other levels of FGA replaced including those fed the control diet (0.0% FGA, *T*_0_). However, the relationship between FCR and FGA levels was expressed by a broken-line model with an identified optimal breakpoint of 23% of FGA inclusion (Figure 2). Increasing the dietary replacement levels of FGA resulted in a gradual decrease in HSI and an increase in SSI and RGL of Nile tilapia (*P* < 0.05).

### 3.2. Endogenous Digestive Enzymes

The data in Table 3 showed that the level of intestinal chymotrypsin, trypsin, lipase, and amylase endogenous enzymes improved in fish fed replaced FGA at levels of 10 and 20%. These values then decreased as the FGA levels increased and in the control (*T*_0_) (*P* < 0.05). The lowest intestinal alkaline phosphatase (ALP) activity was recorded in fish fed the highest replacement level (40%, *T*_4_). No significant differences in ALP were found between fish fed different levels of replacement (10–30%) and the control (*P* > 0.05).

### 3.3. Hematological Parameters

Fish fed FGA levels at 20% and 30% of the daily feed amount had the highest values of Hb and Hct compared to other replacement levels (Table 4). Tilapia fed FGA at 20% had the highest levels of blood indices (i.e., MCH and MCHC) (*P* < 0.05). Furthermore, fish fed FGA at 10% and 20% had significantly higher levels of PLT than those fed other replacement levels (*P* < 0.05). However, no significant differences were detected in the RBC and WBC levels among all treatments (*P* > 0.05).

### 3.4. Serum Biochemical Parameters

No significant differences in the activity of serum ALT, AST, and creatinine were detected among all treatments (Table 5; *P* > 0.05). Meanwhile, serum proteins (TP, ALB, and GLB) and UA increased with increasing levels of FGA when compared to the control (*P* < 0.05).

### 3.5. Antioxidant Enzymes Response

Data in Table 6 showed that increasing levels of FGA led to a significant (*P* < 0.05) decrease in hepatic MDA content compared to those fed FGA at levels of 0% (*T*_0_) and 10% (*T*_1_). The levels of hepatic T-AOC and the activities of CAT and SOD significantly increased with increasing levels of FGA compared to those fed the lowest replacement level of FGA (10%; *T*_1_) and the control (*T*_0_).

### 3.6. Histometric Parameters and Histological Properties of the Intestine

**Figure 3:**
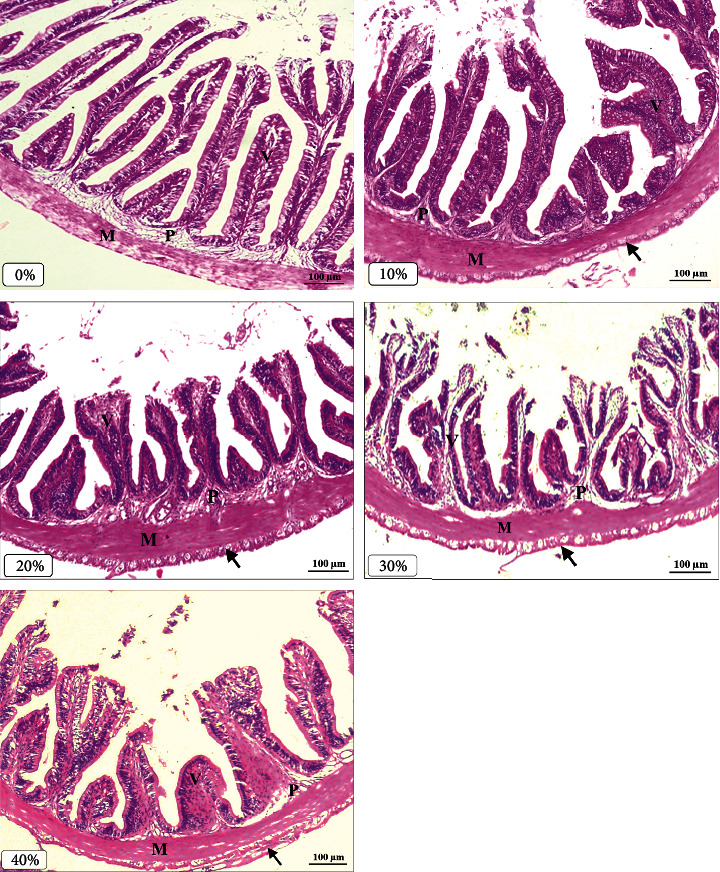
Histomicrograph showing the effects of different levels (0% (*T*_0_), 10% (*T*_1_), 20% (*T*_2_), 30% (*T*_3_), and 40% (*T*_4_)) of fresh green azolla (FGA) on the histological structure of the middle part of the intestine of Nile tilapia. The intestine shows normal histological structures of the intestinal wall and intestinal villi in the control (*T*_0_). Increasing levels of FGA from 10% to 40%, the following histological alterations in the structure of the intestine were observed: increased tunica serosa layer (black arrow), increased thickness of the tunica muscular (M) layer and propria submucosa (P), and shortened length and increased width (×100; bar = 100 *μ*m; H and E stains) of the villi (V).

The histological properties of the intestines are presented in Figure 3 while the intestinal histometric parameters of monosex Nile tilapia fed different levels of FGA are presented in Table 7. Fish fed FGA at levels of 10% (*T*_1_) and 40% (*T*_4_) showed the highest thickness of the mucosa and submucosa layers among all treatments, respectively (*P* < 0.05). Meanwhile, the length and width of the villi decreased significantly in fish fed different levels of FGA compared to the control (*T*_0_). In contrast, the intestinal lumen area increased significantly (*P* < 0.05) by increasing levels of FGA compared to the control.

### 3.7. Chemical Composition of Fish Whole Body and Fillet

The chemical composition of the fish whole body and muscles is shown in Table 8. The fish whole body moisture content significantly increased and crude fat content significantly decreased with increasing levels of FGA (*P* < 0.05). Fish fed FGA at 20% (*T*_2_) had the highest crude protein content and the lowest ash content when compared to other levels (*P* < 0.05). For muscular chemical composition, fish fed FGA at 20% recorded the lowest value of moisture content and the highest values of crude protein and crude fat contents compared to other levels of FGA (*P* < 0.05). However, the highest value of ash content was observed in fish fed FGA at 10% (*T*_1_) (*P* < 0.05).

### 3.8. Flesh Quality

With increasing levels of the dietary FGA, muscular pH, SL, and FLR were significantly decreased (Table 9; *P* < 0.05) compared to the control (*T*_0_). Meanwhile, DL gradually increased as the FGA levels increased until 30% (*T*_3_).

### 3.9. Economic Efficiency

The economic efficiency indicators of Nile tilapia fed different levels of FGA are shown in Table 10. With increasing levels of FGA, there was a significant (*P* < 0.05) decrease in total feed costs and total outputs compared to the control. Fish fed FGA at 0 (*T*_0_) and 20% (*T*_2_) had the highest net return compared to other levels (*P* < 0.05). Furthermore, fish fed 20% FGA had significantly higher economic efficiency (%) than those fed other replacement levels (*P* < 0.05).

## 4. Discussion

In the present study, the growth performance and feed utilization indicated that the fish accepted the replacement of commercial feed with different inclusion levels of FGA, especially at low levels of 10% (*T*_1_) and 20% (*T*_2_). The positive effects of low-level dietary azolla on growth and feed efficiency parameters of Nile tilapia have also been previously reported for the genetically enhanced farmed tilapia (GIFT) strain [23, 25]. More recently, Lumsangkul et al. [47] confirmed positive effects of low-level inclusion of the aquatic fern *Azolla caroliniana* on the growth rate and feed efficiency of treated Nile tilapia. The same beneficial effects of azolla were seen for Thai silver barb, rohu *Labeo rohita*, and fringed-lipped peninsula carp [28, 29, 48]. The high levels of essential amino acids and crude protein found in aquatic weeds like azolla may be responsible for these beneficial effects on growth and feed efficiency in several fish species [49–51]. Additionally, it might be due to the short-chain fatty acid levels in azolla and the increased fish intestinal villi [23, 52]. However, fish fed high replacement levels of FGA up to 20% had significantly lower WG and SGR values. The increased crude fiber content (15.88%), which may cause poor digestion and nutritional absorption, may be linked to the lower growth performance of fish fed high FGA inclusion levels (30% *T*_3_ and 40% *T*_4_). Plant protein sources have large levels of antinutritional factors (ANF), indigestible crude fiber, and carbohydrates [53]. The lack of exogenous enzymes may also explain the poor adoption of FGA as a cheap supplement for fish.

According to Fasakin [54], *Azolla africana* has low digestibility due to its tannin and phytic acid contents. The results are in line with studies by Fasakin et al. [55] and Hossain et al. [56], which replaced fishmeal in the diet of Nile tilapia with haunch (*Sesbania aculeata*) also known as *A. africana*. Additionally, raising the inclusion level of azolla dramatically reduced the SGR of black tiger shrimp, *Penaeus monodon* [57]. Due to the high levels of crude fiber and ANF, azolla may have a negative impact on fish growth performance and feed efficiency [58–60]. Furthermore, it has been discovered that azolla interacts with digestive enzymes that reduce feed utilization and growth efficiency [61]. The SGR of Thai sharpunti (*Barbodes gonionotus*) and Thai silver barb (*Puntius gonionotus* Bleeker) was significantly reduced by a higher azolla inclusion level (75%) in the diet [62, 63]. According to Nekoubin and Sudagar [14], artificially feed-fed grass carp (*Ctenopharyngodon idella*) had a significantly greater FCR than other treatments. Likewise, Das et al. [29] demonstrated that a higher substitution of commercial feed by azolla resulted in decreased protein utilization by Thai silver barb. Ismail et al. [64] recently stated that 30% *Bacillus subtilis* fermented azolla (BSFA) is recommended as a feeding regimen for Nile tilapia for improved growth and feed efficiency parameters. This contrasts with the findings reported in the present study and other studies. The favorable effects of high-level BSFA in comparison to the current study may be attributed to the fermentation of azolla by *B. subtilis*. The fermentation may be linked to the improved feed intake, nutrient digestibility, absorption, and metabolism [5, 65]. Moreover, through fermentation by *B. subtilis*, the BSFA enhanced the immune responses of the fish [66].

The high replacement levels of FGA (up to 20%) considerably reduced the activity of intestinal digestive enzymes such as chymotrypsin, trypsin, lipase, amylase, and ALP. As a result, fish fed high levels of azolla showed poor growth performance, which may be negatively correlated with the reduced activity of the digestive enzymes, feed intake, and digestibility efficiency [67, 68]. The aforementioned digestive enzymes were significantly elevated in tilapia fed low replacement levels of FGA (10% and 20%), which resulted in maximum growth and feed efficiency when compared to tilapia fed FGA levels beyond 20%. Magouz et al. [23] recently discovered that the evaluated endogenous gut enzyme (i.e., amylase, lipase, and protease) activities of GIFT strains were not substantially altered by dietary azolla. Additionally, GIFT strains fed a diet comprising a low amount of azolla (15%) and 3% *A. platensis* as a fishmeal substitute had no discernible effects on the same digestive enzyme activities [25]. The diverse types of dietary azolla, their quantities, sources, experimental time, and management may also be factors in the variations between our findings and other studies.

An essential role is played by physiological indices that measure hematological and serum biochemical markers which help in identifying potential nutritional impacts on fish health [69, 70]. Hematological and biochemical markers in the current investigation indicated nearly normal results. These findings demonstrated that FGA has no negative effects on fish health. Additionally, feeding FGA at modest doses of 10% (*T*_1_) and 20% (*T*_2_) considerably increased Hb, Hct, blood indices, and PLT values, which show that the fish were anemia-free [23]. In diets containing 15% azolla and 3% *A. plantenis*, AST and ALT activities were markedly decreased compared to the control and the GIFT strain fed azolla [25]. Biopolymers, vitamins A, C, and B12, beta-carotene, and essential minerals in azolla were able to stop oxidative damage in fish cells or tissues and were thought to act as protective agents for fish that had been treated [71]. In this context, minerals in aquatic plants are crucial for metabolic functions and cell movement. They also serve as cofactors for catalytic enzymes [72].

The functional status of the antioxidant system and antioxidant enzymes reflects the ability of the body to digest free radicals and protect tissues from oxidative damage [70]. The current findings demonstrated that dietary FGA up to a 20% replacement of commercial feed considerably increased the antioxidant responses of Nile tilapia, indicating less cell damage in the treated fish. This may be caused by the presence of the chemical C-phycocyanin, which has potent antiarthritic, anti-inflammatory, neuroprotective, and hepatoprotective properties. These properties are strongly related to its antioxidative activity and maintenance of the immune system of fish [73]. These results are in line with those of Mosha et al. [25], who found that adding azolla to water greatly increased the catalase, superoxide dismutase, and glutathione peroxidase activities in GIFT strains. There is substantial antioxidant activity in the algal carotenoid extracts [74].

The height and width of the villi as well as the quantity of goblet cells are intestinal morphometrics that can be used to forecast absorption mechanisms in fish [75, 76]. With higher levels of substituted FGA up to 20%, the villous width and length of Nile tilapia dramatically decreased in the current study. The gut lumen area and mucosa and submucosa layer thicknesses were reported to be maximum in the high inclusion levels of FGA. Caspary [77] showed that increased villi surface areas can enhance nutrient absorption. The high quantities of crude protein and essential amino acids that may increase intestinal absorptive capacity, lessen scours, and promote fish growth may be linked to the improvement of growth and feed utilization in Nile tilapia fed low levels of FGA [50, 67]. The increased goblet cell numbers in the intestines of GIFT strains fed azolla suggest that the fish may have better immunity and nutritional value [23]. On the other hand, Mello et al. [78] demonstrated that fish fed azolla had increased intestinal cells, which boosted their metabolism and absorption of nutrients. Overall, the capacity of tilapia to digest feed more effectively was positively impacted by azolla, as shown in the improved growth performance. The goblet cells may also shield the mucosal layer from dehydration, pathogens, and injury by expelling mucus, as well as from antibacterial agents and pathogens that can harm the fish [79, 80].

The chemical composition of the fish whole body is influenced by the type and composition of the feed. The chemical composition of the fish fillets, which determines the nutritional quality [22, 29, 81], demonstrated that high levels of azolla in fish diets were inversely related to body protein and fat. In this study, the chemical makeup of the entire fish and muscle showed the same pattern. The findings showed that increasing levels of FGA were inversely correlated with body protein and fat. These results are consistent with the beneficial effects of FGA on growth efficiency and feed consumption. Azolla includes various bioactive substances that support fish growth muscles including carotenoid, chlorophyll, beta-carotene, vitamin E, and minerals (i.e., potassium and iron) [82, 83]). This improvement might be explained by the higher levels of digestive enzymes that were found to boost nutritional utilization and absorption. Al-Aaraji and Taha [27] discovered that azolla supplementation decreased the amount of fat in common carp muscles. However, Magouz et al. [23] reported that the incorporation of azolla in tilapia diets had no influence on the body proximate analysis.

To our knowledge, no prior research has examined how dietary azolla affects the quality of their meat. The total carotenoid concentration in the muscle tissues of GIFT strains was recently examined by Mosha et al. [25] as a gauge for customer acceptance of fish products. They discovered that as azolla levels rose, so did the total carotenoid content in the fish muscles. With the increased amounts of dietary FGA, muscle pH, SL, and FLR significantly decreased, indicating an improvement in flesh quality. This may be related to the enhanced chemical composition (i.e., increased protein and decreased fat) due to the increased ability of the muscle to hold water. According to Bjørnevik and Solbakken [84], water loss from fish muscles is unsuitable for human consumption or commercial use.

## 5. Conclusions

Based on the current findings, among all dietary treatments, a replacement level of 20% (*T*_2_) FGA or lower could positively affect the growth, feed utilization, and intestinal endogenous enzymes as well as ameliorate hematobiochemical parameters, oxidative responses, and flesh quality of Nile tilapia. Long-term studies are needed to potentially determine the optimum level of FGA for tilapia. Further studies are required to understand the effects of dietary FGA as an unconventional strategy on the physiology, immune responses, comparative endocrine, and oxidative status of fish reared under different environmental or management conditions for a sustainable commercial aquaculture.

## Figures and Tables

**Table 1:** Chemical analysis of the basal diet (control) and fresh green azolla (% on dry matter basis).

Nutrient composition	Basal diet	Fresh green azolla
Dry matter (DM)	91.73	10.22
Crude protein (CP)	25.25	24.97
Ether extract (EE)	5.15	3.21
Ash	5.32	10.76
Crude fiber (CF)	4.35	15.88
Nitrogen free extract (NFE)^a^	59.93	45.18
Gross energy (MJ 100 g^−1^ DM) (GE)	1826	1490

Floating basal feed was purchased from Grand Aqua Manufactory for Fish Feed, Damietta Government, Egypt. ^a^NFE = 100 − (CP + EE + CF + Ash). ^b^GE (MJ 100 g^−1^ DM) = (CP × 23.64) + (EE × 39.54) + (NFE × 17.11).

**Table 2:** Effect of the different levels of fresh green azolla (FGA) on growth performance, feed efficiency, and body indices of adult Nile tilapia males.

Parameters	FGA level (%)					±PSE	*P* value
	0 (*T*_0_)	10 (*T*_1_)	20 (*T*_2_)	30 (*T*_3_)	40 (*T*_4_)		
FW (g)	207.0^c^	221.0^b^	229.0^a^	199.3^d^	177.3^e^	0.918	0.0001
TWG (g)	98.50^c^	113.0^b^	120.9^a^	91.30^d^	69.17^e^	0.901	0.0001
ADG (g fish^–1^ day^–1^)	1.41^c^	1.61^b^	1.73^a^	1.30^d^	0.99^e^	0.012	0.0001
SGR (% day^–1^)	0.93^c^	1.02^b^	1.07^a^	0.88^a^	0.71^e^	0.006	0.0001
FCR	2.01^a^	1.62^c^	1.43^d^	1.73^b^	2.07^a^	0.023	0.0001
PER	1.92^d^	2.39^b^	2.69^a^	2.23^c^	1.86^d^	0.026	0.0001
HSI (%)	3.94^a^	3.33^ab^	3.70^a^	3.22^ab^	2.79^b^	0.210	0.004
SSI (%)	0.71^c^	1.09^a^	0.57^d^	1.00^ab^	0.91^b^	0.080	0.0002
RGL	4.45^d^	4.94^b^	4.72^c^	4.92^b^	5.12^a^	0.253	0.0203

Means in the same row having different superscripts are significantly different (*P* < 0.05). The values are shown as mean; PSE: pooled standard error; FW: final body weight; TWG: total weight gain; ADG: average weight gain; SGR: specific growth rate; FCR: feed conversion ratio; PER: protein efficiency ratio; HSI: hepatosomatic index; SSI: stomach somatic index; RGL: relative gut length.

**Table 3:** Effect of different levels of FGA on the intestinal endogenous enzymes of Nile tilapia.

Parameter (U g^–1^ tissue)	FGA level (%)					±PSE	*P* value
	0 (*T*_0_)	10 (*T*_1_)	20 (*T*_2_)	30 (*T*_3_)	40 (*T*_4_)		
Chymotrypsin	5.56^f^	10.65^a^	9.36^a^	8.90^c^	6.89^d^	0.25	0.001
Trypsin	0.78^b^	0.91^a^	0.89^a^	0.77^b^	0.72^b^	0.014	0.012
Lipase	913^b^	1000^a^	990^b^	900^c^	896^d^	15.13	0.035
Amylase	712^b^	730^a^	720^a^	700^c^	690^d^	14.12	0.023
Alkaline phosphatase	31.58^a^	35.12^a^	31.98^a^	30.18^a^	28.13^b^	0.56	0.032

Means followed by different superscripts in the same row are significantly different (*P* < 0.05). The values are shown as mean; PSE: pooled standard error.

**Table 4:** Effect of different levels of FGA on the hematological parameters of Nile tilapia.

Parameters	FGA level (%)					±PSE	*P* value
	0 (*T*_0_)	10 (*T*_1_)	20 (*T*_2_)	30 (*T*_3_)	40 (*T*_4_)		
Hb (g dL^–1^)	7.68^ab^	7.55^ab^	8.45^a^	8.45^a^	7.00^b^	0.38	0.007
Hct (%)	28.98^ab^	28.79^ab^	30.20^ab^	32.42^a^	27.77^b^	0.87	0.018
RBCs (×10^6^ mm^–3^)	2.00	2.03	2.13	2.27	2.03	0.08	0.174
MCV (*μ*^3^)	144.9^a^	135.7^b^	136.4^b^	145.3^a^	134.9^b^	0.79	0.001
MCH (*μ*^3^)	38.00^ab^	40.17^a^	39.65^a^	37.83^b^	36.43^b^	0.44	0.002
MCHC (%)	26.35^b^	27.77^ab^	29.35^a^	26.25^b^	26.63^b^	0.53	0.004
WBCs (×10^3^ mm^–3^)	125.0	121.2	121.6	129.3	124.6	2.41	0.185
PLT (×10^3^ mm^–3^)	149.0^b^	164.3^a^	159.0^a^	141.7^b^	136.7^c^	4.82	0.005

Means in the same column having different superscripts are significantly different (*P* < 0.05). The values are shown as mean; PSE: pooled standard error; Hb: hemoglobin; PCV: packed cell volume; RBCs: red blood cells; MCV: mean corpuscular volume; MCH: mean corpuscular hemoglobin; MCHC: mean corpuscular hemoglobin concentration; WBCs: white blood cells; and PLT: blood platelets.

**Table 5:** Effect of different levels of the FGA on the serum biochemical parameters of Nile tilapia.

Parameter	FGA level (%)					±PSE	*P* value
	0 (*T*_0_)	10 (*T*_1_)	20 (*T*_2_)	30 (*T*_3_)	40 (*T*_4_)		
*Liver function parameters*							
ALT (U L^–1^)	3.50	4.50	4.00	5.00	3.25	0.52	0.237
AST (U L^–1^)	5.33	4.00	5.50	4.20	5.00	0.48	0.220
TP (g dL^–1^)	2.14^b^	2.98^b^	3.35^a^	3.53^a^	3.51^a^	0.27	0.025
ALB (g dL^–1^)	1.06^b^	1.25^a^	1.31^a^	1.20^ab^	1.15^ab^	0.04	0.029
GLB (g dL^–1^)	1.17^b^	2.13^a^	2.21^a^	2.33^a^	2.04^a^	0.14	0.001

*Kidney function parameters*							
Creatinine (mg dL^–1^)	0.53	0.73	0.71	0.68	0.75	0.07	0.252
UA (mg dL^–1^)	0.57^c^	0.50^c^	0.53^c^	0.75^b^	1.05^a^	0.05	0.005

*Thyroid hormones*							
T3 (ng dL^–1^)	129.3	126.4	126.7	126.1	122.5	1.73	0.171
T4 (*μ*g dL^–1^)	2.30	2.20	2.43	2.37	2.43	0.10	0.461

Means followed by different superscripts in the same row are significantly different (*P* < 0.05). The values are shown as mean; PSE: pooled standard error; ALT: alanine transaminase; AST: aspartate transaminase; TP: total protein; ALB: albumin; GLB: globulin; UA: uric acid; T3: triiodothyronine; T4: thyroxine.

**Table 6:** Effect of different levels of FGA on the hepatic antioxidant parameters of Nile tilapia.

Parameter (U g^–1^ protein)	FGA level (%)					±PSE	*P* value
	0 (*T*_0_)	10 (*T*_1_)	20 (*T*_2_)	30 (*T*_3_)	40 (*T*_4_)		
MDA	73.10^a^	57.50^b^	48.50^c^	49.00^c^	51.02^c^	0.311	0.0301
CAT	10.40^c^	13.40^b^	14.10^a^	14.55^a^	14.35^a^	0.540	0.0460
SOD	68.00^c^	68.20^b^	101.10^a^	103.10^a^	97.23^a^	1.28	0.0132
T-AOC	21.40^b^	34.45^ab^	37.05^a^	38.65^a^	39.33^a^	1.25	0.0253

Means followed by different superscripts in the same row are significantly different (*P* < 0.05). The values are shown as mean; PSE: pooled standard error; MDA: malondialdehyde; CAT: catalase; SOD: superoxide dismutase; T-AOC: total antioxidant capacity.

**Table 7:** Effect of different levels of FGA on the intestinal histometric parameters of Nile tilapia.

FGA level (%)	Muscular (*μ*m)	Submucosa (*μ*m)	Intestinal villi		Intestine lumen area (*μ*m^2^)
			Length (*μ*m)	Width (*μ*m)	
0 (*T*_0_)	11.18^c^	9.36^b^	77.94^a^	18.49^a^	7002^c^
10 (*T*_1_)	15.47^a^	9.01^b^	48.38^b^	14.56^bc^	26938^b^
20 (*T*_2_)	12.16^b^	9.06^b^	46.88^b^	15.56^b^	20394^b^
30 (*T*_3_)	12.91^b^	9.69^b^	43.03^b^	13.82^c^	42447^a^
40 (*T*_4_)	13.01^ab^	10.85^a^	44.69^b^	17.62^ab^	55578^a^
±PSE	0.582	0.442	1.730	0.422	3354
*P* value	0.0001	0.0219	0.0001	0.0001	0.0001

Means followed by different superscripts in the same column are significantly different (*P* < 0.05). The values are shown as mean; PSE: pooled standard error.

**Table 8:** Effect of different levels of FGA on the chemical composition of the whole body and muscle of Nile tilapia.

Item	FGA level (%)					±PSE	*P* value
	0 (*T*_0_)	10 (*T*_1_)	20 (*T*_2_)	30 (*T*_3_)	40 (*T*_4_)		
*Chemical composition of the whole body*							
Moisture (%)	76.26^c^	77.27^b^	75.16^d^	78.15^ab^	78.63^a^	0.233	0.0001
Crude protein (%)	14.90^b^	14.39^b^	17.07^a^	13.10^c^	13.69^c^	0.123	0.0001
Crude fat (%)	5.13^a^	4.28^b^	4.69^b^	4.80^b^	3.93^c^	0.060	0.0001
Ash (%)	3.72^b^	4.06^a^	2.82^c^	3.95^ab^	3.75^b^	0.055	0.0001

*Chemical composition of the fish muscle*							
Moisture (%)	83.50^a^	83.89^a^	82.98^b^	83.68^a^	83.44^a^	0.071	0.0001
Crude protein (%)	14.65^b^	14.36^b^	15.14^a^	14.77^b^	14.72^b^	0.062	0.0001
Crude fat (%)	0.96^a^	0.75^c^	0.95^a^	0.58^d^	0.90^b^	0.010	0.0001
Ash (%)	0.85^d^	1.01^a^	0.88^c^	0.95^b^	0.95^b^	0.020	0.0022

Means followed by different superscripts in the same column are significantly different (*P* < 0.05). The values are shown as mean; PSE: pooled standard error.

**Table 9:** Effect of different levels of FGA on the flesh quality of adult Nile tilapia.

Parameters	FGA level (%)					±PSE	*P* value
	0 (*T*_0_)	10 (*T*_1_)	20 (*T*_2_)	30 (*T*_3_)	40 (*T*_4_)		
pH	7.49^a^	7.41^a^	6.78^b^	6.84^b^	6.93^b^	0.043	0.001
SL (%)	2.44^a^	2.01^c^	2.11^b^	1.75^d^	1.54^e^	0.157	0.003
DL (%)	4.10^c^	4.33^b^	4.65^a^	4.61^a^	4.05^c^	0.210	0.016
FLR (%)	0.707^a^	0.505^c^	0.573^c^	0.538^c^	0.668^b^	0.048	0.025

Means in the same row having different superscripts are significantly different (*P* < 0.05). The values are shown as mean; PSE: pooled standard error; SL: stored loss (%); DL: drip loss (%); FLR: frozen leakage rate (%).

**Table 10:** Effect of different levels of FGA on the economic efficiency of Nile tilapia.

Parameters	FGA level (%)					±PSE	*P* value
	0 (*T*_0_)	10 (*T*_1_)	20 (*T*_2_)	30 (*T*_3_)	40 (*T*_4_)		
Total feed costs ($)^1^	27.70^a^	25.60^b^	24.20^c^	22.10^d^	20.10^e^	0.180	0.0001
Total outputs ($)^2^	42.37^a^	34.44^c^	39.51^b^	31.92^d^	24.18^e^	0.299	0.0001
Net return ($)^3^	14.65^a^	8.87^b^	15.27^a^	9.78^b^	4.13^c^	0.347	0.0001
Economic efficiency (%)^4^	52.93^b^	34.68^d^	63.03^a^	44.20^c^	20.59^e^	1.63	0.0001

Means followed by different superscripts in the same column are significantly different (*P* < 0.05). The values are shown as mean; PSE: pooled standard error. ^1^Total costs per treatment ($) = total cost of the commercial diet + total cost of fresh azolla. ^2^Total outputs ($/Kg) = fish price × total fish production (kg). ^3^Total net return ($) = total output–total costs. ^4^Economic efficiency (%) = (net return/total costs) × 100. (According to the Egyptian market, the price of feed was 10.00 LE kg^–1^; the price of fresh azolla was 1.00 LE kg^–1^; and fish prices were 25.00 LE kg^–1^; 1 American dollar = 16.09 Egyptian pounds at the time that this experiment was carried out).

## Data Availability

Data is available upon request.
